# Preoperative dual-energy X-ray absorptiometry and FRAX in patients with lumbar spinal stenosis

**DOI:** 10.1186/s13018-018-0964-1

**Published:** 2018-10-16

**Authors:** Camilla Bergh, Ann-Charlott Söderpalm, Helena Brisby

**Affiliations:** 1 Department of Orthopaedics, Institute of Clinical Sciences, Sahlgrenska Academy, Gothenburg University, Gothenburg, Sweden; 2 Department of Orthopaedics, Sahlgrenska University Hospital, SE-413 45 Gothenburg, Sweden

**Keywords:** Lumbar spinal stenosis, DXA, FRAX, Osteoporosis, Bone mineral density

## Abstract

**Background:**

Osteoporosis implies an increased risk of complications after orthopedic surgery. For the mostly elderly group of patients undergoing lumbar spinal stenosis surgery (LSS), it is important to include skeletal health evaluation in the preoperative planning. The aim of this study was to assess spine and femoral neck (FN) bone mineral density (BMD) in LSS patients and to evaluate whether the World Health Organization (WHO) fracture risk assessment tool (FRAX) can identify patients with reduced BMD levels in the spine.

**Methods:**

The study involved 65 LSS patients and 53 patients with hip osteoarthritis (HOA) for comparison. BMD was measured with dual-energy X-ray absorptiometry (DXA) in the lumbar spine in three projections: anterior-posterior (AP), lateral and lateral-mid (the mid-portion of the vertebrae), and FN. The LSS patients filled out the FRAX questionnaire.

**Results:**

In the LSS group, 43% of the women and 8% of the men were classified as being osteoporotic/osteopenic by AP spine measurement. The corresponding proportions using the lateral spine *T*-score ≤ − 2.5 were 87% and 57%, respectively, and 82% and 53%, respectively, for the FN. The FN BMD *T*-score was significantly lower in the LSS group compared with the HOA group. The FRAX questionnaire identified 40% of the LSS patients with a moderate/high risk of sustaining an osteoporotic fracture within 10 years, with or without simultaneous FN BMD, while 71% of these patients were classified as being osteoporotic with DXA lateral spine measurement.

**Conclusion:**

It is common with osteoporosis/osteopenia in patients undergoing spine surgery, and the identification may influence the surgical treatment why the evaluation of BMD is important preoperatively. FRAX evaluation could not replace DXA measurement of the spine regarding the identification of osteoporosis patients in the preoperative planning phase.

## Background

Degenerative lumbar spinal stenosis (LSS) is the most common indication for lumbar surgery [1]. It is treated with decompression, with or without fusion, when surgery is indicated. Most of the patients who suffer from spinal stenosis are 65 years of age or older [2, 3].

Symptoms of LSS often lead to limited physical activity including walking or prolonged standing [4, 5], mostly because of leg and/or back pain [5–7]. Osteoporosis and osteopenia are conditions that become more pronounced with age [8], and they are well known to be influenced by immobilization and low physical activity [6]. It has previously been reported that women over 50 years of age who undergo spine surgery have a high incidence of osteopenia and osteoporosis: 44.1% and 51.3%, respectively [9]. Furthermore, osteoporosis and osteopenia are associated with a higher risk of complications such as screw-loosening and fractures in end-instrumented vertebrae [10–13]. The knowledge of a low BMD may also influence the surgical methods for these patients; if instrumentation is used, augmentation may be considered [14].

Despite the demonstrated association between bone mineral density (BMD) and an increased risk of complications postoperatively [12], preoperative planning in spine surgery patients does not always include bone quality evaluation. Dual-energy X-ray absorptiometry (DXA) [15] is the method that is most often used to measure the BMD. A gender- and ethnic group-matched *T*-score is calculated and used for the evaluation of osteoporosis/osteopenia [16]. BMD can be measured in different projections and locations, such as anterior-posterior (AP) or lateral projections of the lumbar spine and anterior-posterior projection of the femoral neck (FN). The projection that is best for evaluating the overall, as well as local, bone quality may vary in different patient groups [17, 18]. For example, due to facet joint osteoarthritis/osteophytes, which are common in LSS patients, AP lumbar measurements may overestimate the BMD relative to the lateral lumbar projection [19, 20]. Another frequently used method for indirect evaluation of bone quality is the World Health Organization (WHO) fracture risk assessment tool (FRAX), which focuses on the prediction of future fracture risk. This questionnaire can be used with or without FN BMD [16, 21]. Another method to measure BMD is quantitative computed tomography (QTC) which exposes the patient to an increased radiation dose compared to DXA [22].

The aim of the present study was to investigate differences in frequency of osteoporosis/osteopenia, as measured by DXA in different locations and different projections, in a cohort of patients planned for LSS surgery. An age- and gender-matched group of hip osteoarthritis (HOA) patients (with similar anticipated pain and immobilization problems) was used for comparison. Furthermore, we investigated the value of using the FRAX questionnaire, either alone or in combination with FN BMD, to identify spine patients with lumbar osteoporosis/osteopenia. The rationale behind this is that the FRAX can easily be obtained and there is often an FN BMD measurement at the time of referral in LSS patients.

## Methods

### Patient population

Patients over 50 years of age, who were planned for first-time surgery because of LSS or HOA, were prospectively included in the study during the periods 2013–2014 and 2016 (due to the reorganization of the DXA unit, no patients were included during 2015). Sixty-five patients diagnosed with LSS and of 53 patients with HOA were included in the study. The LSS patients were all planned for decompressive surgery alone or in combination with an instrumented fusion. All HOA patients were planned for total hip replacement. Exclusion criteria were previous surgery (hip or spine) and ongoing medical treatment for osteoporosis and/or rheumatoid arthritis. The study was approved by the regional ethical review board in Gothenburg, Sweden (reference number: 104-11). All the study participants gave their written informed consent.

### Dual-energy X-ray absorptiometry (DXA)

All patients underwent bone densitometry with DXA for assessment of BMD 1–2 weeks before surgery. All measurements were performed by the same investigator using the Hologic Discovery™ densitometer (Hologic, Bedford, MA, USA).

BMD was measured in four different areas/projections of the spine and hip: (1) AP lumbar spine projection including the second to the fourth lumbar vertebrae (L2-L4), with separate measurement values for each vertebra; (2) lateral lumbar spine projection (lateral) including L1–L4, with separate measurement values for each vertebra; (3) lateral-middle lumbar spine projection (lat-mid), a smaller area in the middle part of each vertebra in the lateral projection; and (4) the AP femoral neck projection (FN).

*T*-score values were calculated for all BMD measurements and used for classification of osteoporosis/osteopenia. The WHO definitions for osteoporosis and osteopenia were used, i.e., osteoporosis: *T*-score ≤ − 2.5; osteopenia: − 1 > *T*-score > − 2.5; and normal: *T*-score ≥ − 1 [16]. This definition has been calculated based on AP spine and FN measurements.

The *Z*-score was calculated for all measurements and used for comparison between the two groups, since this value compensates for age, gender, and ethnicity.

### WHO fracture risk assessment tool (FRAX)

All patients completed the FRAX questionnaire [23], which includes questions on previous fragility fractures, rheumatoid arthritis, smoking habits, steroid use, alcohol usage, and heredity for hip fractures. Together with information on age, BMI, and gender, a score is created predicting the risk of fractures, such as clinical spine, wrist, proximal humerus, and hip, during the coming 10 years. This score can be calculated with or without the inclusion of FN BMD.

The FRAX questionnaire alone or in combination with DXA FN was compared to the DXA lumbar lateral vertebral measurements. The lumbar lateral vertebral measurements were here considered the best available reference for vertebral bone quality based on previous reports [19].

### Statistics

All results are reported as mean ± SD unless otherwise stated. For group comparisons, Student’s *t* test was used and a significance level of *p* ≤ 0.05 was used*.* Absolute 10-year fracture risk probabilities using the FRAX model alone and FRAX with FN BMD were categorized as low-risk (< 10%), moderate risk (10–20%), and high-risk (> 20%) [24]. Statistical analysis was performed using the IBM SPSS 22.0 software (IBM Corp., Armonk, NY, USA).

## Results

### Patient characteristics

The patient characteristics are described in Table 1.

**Table 1:** Gender, age, and BMI in LSS and HOA patients

	LSS		HOA	
	Female	Male	Female	Male
Gender, *n* (%)	37 (57)	28 (43)	33 (62)	20 (38)
Age, years ± SD	68 ± 8	66 ± 9	70 ± 8	70 ± 8
BMI, kg/m2 ± SD	28 ± 4	28 ± 4	27 ± 5	26 ± 3

#### Lumbar spinal stenosis patients

##### BMD in different locations/projections

LSS patients generally had higher AP BMD relative to lateral and lat-mid BMD, for the aggregated total lumbar measurement and for single lumbar vertebrae levels (Table 2). In this patient group, FN BMD values were generally between the AP BMD levels and the lateral BMD spine levels (Table 3).

**Table 2:** BMD, *T*-score, and *Z*-score in LSS patients planned for surgery

Lumbar level	BMD			*T*-score			*Z*-score		
	AP	Lateral	Lateral mid	AP	Lateral	Lateral mid	AP	Lateral	Lateral mid
L2	1.023 ± 0.203	0.626 ± 0.143 ***	0.527 ± 0.163 ***	− 0.1 ± 1.8	− 2.3 ± 1.4 ***	− 2.7 ± 1.2 ***	1.7 ± 1.9	0.6 ± 1.5 ***	0.2 ± 1.2 ***
L3	1.049 ± 0.208	0.661 ± 0.152 ***	0.573 ± 0.190 ***	0.1 ± 1.9	− 2.2 ± 1.7 ***	− 2.0 ± 1.7 ***	2.0 ± 2.0	0.7 ± 1.8 ***	0.5 ± 1.8 ***
L4	1.138 ± 0.202	0.670 ± 0.197 ***	0.628 ± 0.229 ***	0.7 ± 1.8	− 1.1 ± 2.0 ***	− 1.6 ± 1.8 ***	2.6 ± 1.9	1.6 ± 2.3 ***	0.9 ± 2.0 ***
total	1.056 ± 0.186	0.682 ± 0.135 ***	0.578 ± 0.140 ***	0.5 ± 1.7	− 1.7 ± 1.6 ***	− 2.2 ± 1.3 ***	1.9 ± 1.8	1.7 ± 2.0	1.0 ± 1.8 ***

BMD, *T*-score, and *Z*-score for lumbar spinal stenosis patients (LSS) in three different projections, anterior-posterior (AP), lateral, and lateral mid

Results are shown for each single vertebra (L2, L3, L4) and also for these vertebrae combined measurements in different projections for single vertebrae differed significantly when comparing AP with lateral and AP with lat-mid. ***p < 0.001

**Table 3:** Total BMD in LSS patients

	Total BMD LSS	
	Female	Male
AP	1.01 (±0.18)	1.12 (±0.17) *
Lateral	0.64 (±0.14)	0.74 (±0.11) **
Lateral mid	0.56 (±0.16)	0.51 (±0.11)
FN	0.71 (±0.11)	0.76 (±0.11)

Total BMD for lumbar spinal stenosis patients (LSS) in four different projections: anterior-posterior (AP), lateral, lateral mid, and femoral neck (FN). Comparison between women and men

*p < 0.05, **p < 0.01

There was a significant difference between men and women in the LSS group regarding total AP BMD (1.12 ± 0.17 g/cm^2^ and 1.01 ± 0.18 g/cm^2^, respectively; *p* = 0.016) and for lateral BMD (0.74 ± 0.11 g/cm^2^ and 0.64 ± 0.14 g/cm^2^; *p* = 0.003). There were no significant differences between men and women regarding lat-mid BMD (0.51 ± 0.11 g/cm^2^ and 0.56 ± 0.16) g/cm^2^, respectively; *p* = 0.303) or FN BMD (0.76 ± 0.11 g/cm^2^ and 0.71 ± 0.11 g/cm^2^; *p* = 0.066).

##### *T*-score in different locations/projections and rate of osteoporosis/osteopenia

For the LSS patients, the mean *T*-score in the lumbar spine AP projection was 0.1 ± 1.7 as compared to − 1.6 ± 1.6 in the lateral projection (*p* < 0.001). *T*-score for the lat-mid measurements was even lower at − 2.2 ± 1.3 (Table 4).

**Table 4:** *T*-score and *Z*-score in LSS and HOA patients planned for surgery

	*T*-score				*Z*-score			
	AP	Lateral	Lateral mid	FN	AP	Lateral	Lateral mid	FN
LSS	0.1 (± 1.7)	− 1.6 (± 1.6)	− 2.2 (± 1.3)	0.7 (± 0.1)	1.9 (± 1.8)	1.7 (± 2.0)	1.0 (± 1.8)	0.5 (± 1.0)
HOA	− 0.6 (± 1.7)	− 1.3 (± 2.3)	− 2.0 (± 1.7)	0.8 (± 0.1)	1.9 (± 1.7)	2.4 (± 2.3)	1.6 (± 1.8)	1.2 (± 1.6)
*p* value	0.51	0.38	0.68	*	0.86	0.11	0.74	*

*Z*-score and *T*-score for lumbar spinal stenosis (LSS) and hip osteoarthrosis (HOA) patients in four different projections: anterior-posterior (AP), lateral, lateral mid, and femoral neck (FN). Comparison between LSS and HOA patients for the different projections. *p < 0.05

In the present study using the WHO cutoff for osteoporosis/osteopenia, the *T*-scores for the AP projection (L2–L4) meant that 5% of the women were classified as being osteoporotic and 38% were classified as being osteopenic. Using total lateral spine projection, altogether, 87% were classified as having *T*-score < − 1. The proportion was similar for the lat-mid projection measurements. The FN measurements led to the classification of 14% of the LSS women as being osteoporotic and 68% as being osteopenic (Table 5).

**Table 5:** The presence of osteoporosis and osteopenia in LSS and HOA patients planned for surgery

	LSS				HOA			
	AP %	Lateral %	Lateral mid %	FN %	AP %	Lateral %	Lateral mid %	FN %
	female/men	female/men	female/men	female/men	female/men	female/men	female/men	female/men
*T*-score ≤ − 2.5	5/4	49/11	49/46	14/2	12/0	44/12	46/24	13/0
− 1 > *T*-score > − 2.5	38/4	38/46	40/36	68/51	36/10	28/35	36/47	47/47
*T*-score ≥ − 1	57/92	13/43	11/18	18/47	52/90	28/53	18/29	40/53

Osteoporosis defined as *T*-score ≤ − 2.5; osteopenia: − 1 > *T*-score > − 2.5; and normal: *T*-score ≥ − 1. All values in tables are percentages within each projection, subdivided according to gender. Four different projections: anterior-posterior (AP), lateral, lateral mid, and femoral neck (FN)

The proportion of men classified through AP projection as having osteoporosis or osteopenia was only 8% in total. Using the lateral spine projection, the percentage of men having a *T*-score < − 1 was still much lower than for women (57% as opposed to 87%). However, the number of patients having a *T*-score < − 1 using the lat-mid projection was relatively similar in men and women (82% and 89%, respectively) (Table 5).

**Fig. 1:**
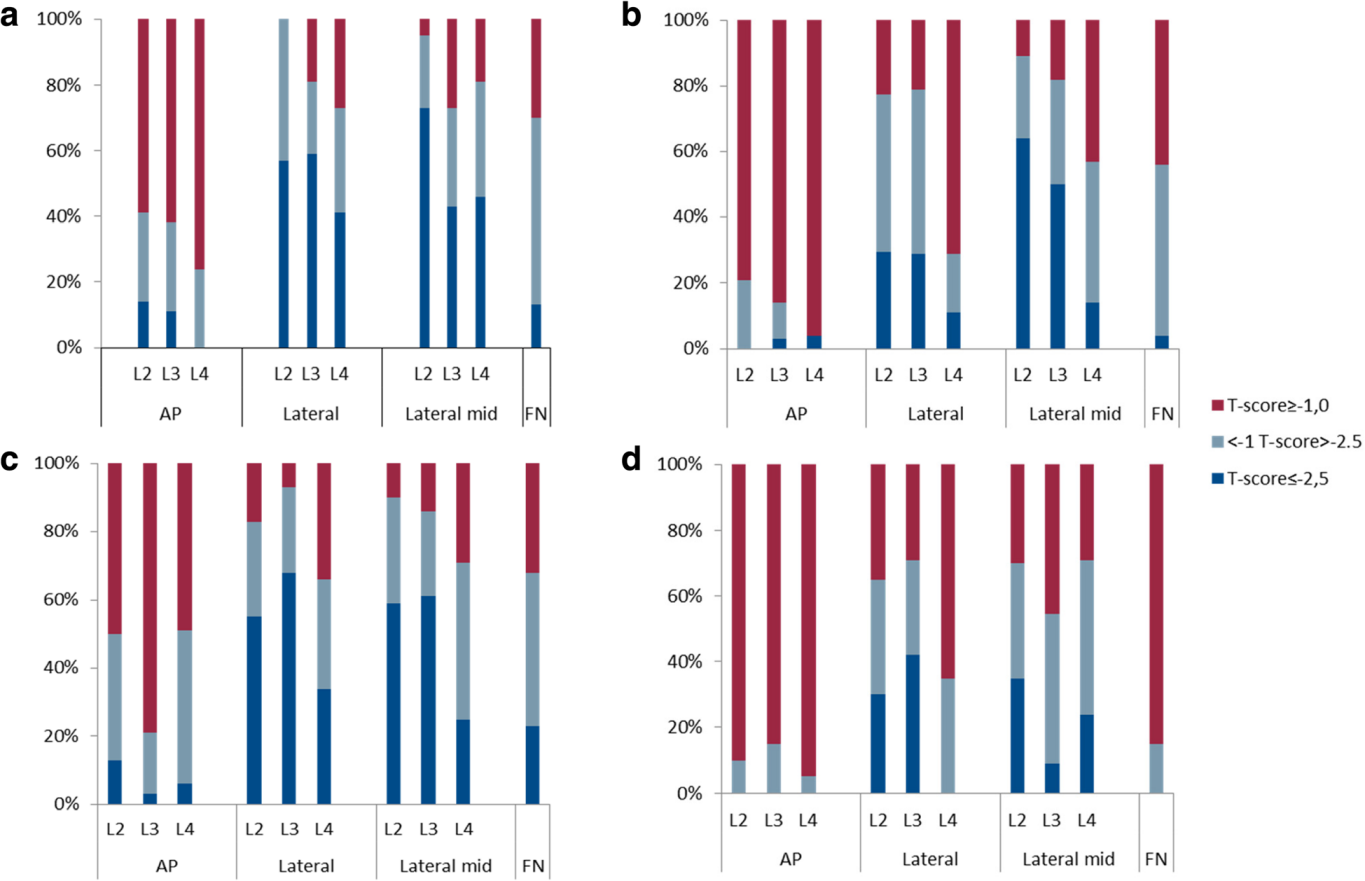
Percentage of patients classified as having osteoporosis, osteopenia, or normal bone quality in **a** women with LSS (*n* = 37), **b** men with LSS (*n* = 28), **c** women with HOA (*n* = 33), and **d** men with HOA (*n* = 20). The *T*-score was based on DXA measurements in four different/projections: lumbar spine anterior-posterior (AP), lateral spine (lateral), lateral-mid spine (lateral mid), and the femoral neck (FN). Osteoporosis: *T*-score ≤ − 2.5; osteopenia: − 1 > *T*-score > − 2.5; and normal: *T*-score ≥ − 1

When we compared the *T*-score for one single lumbar vertebra level (L2–L4), for both men and women and for all projections, there were increasing numbers with values < − 1 when more caudal measurements were performed (Fig. 1a, b).

#### Lumbar spinal stenosis patients compared to hip osteoarthritis patients

##### Comparison of BMD values between patient groups

No significant differences were found between LSS patients and HOA patients regarding the lumbar BMD values, either in the AP projection (1.05 ± 0.19 and 1.03 ± 0.19, respectively; *p* = 0.252) or in the lateral projection (0.69 ± 0.20 and 0.63 ± 0.53; *p* = 0.289). For single vertebra levels, only the AP for the L4 vertebra showed a slight difference at the group level: LSS 1.14 ± 0.20 and HOA 1.06 ± 0.20 (*p* = 0.033). Furthermore, there was a small difference in mean BMD for the FN between the two groups: LSS 0.72 ± 0.14 and HOA 0.77 ± 0.13 (*p* = 0.028).

##### Comparison of *T*-score and *Z*-score between the two patient groups

The comparison of the AP spine projection *T*- or *Z*-scores between the two patient groups did not reveal any significant differences nor did the lateral spine projection or the lat-mid measurements for three vertebrae show any significant differences between the two groups regarding *T*- or *Z*-score. For the FN measurements, however, both the *T*-score and the *Z*-score were significantly higher in the HOA group than in the LSS group (Table 4).

The lat-mid spine projection demonstrated more patients as having *T*-score ≤ **−** 2.5 than any other projection, regardless of gender or diagnosis. Using the FN projection, fewer patients were classified as having osteoporosis, i.e., less than 15% of the women in both the LSS group and the HOA group and no men in either of the groups (Table 5). The distribution of osteoporosis and osteopenia in the LSS and HOA groups showed a similar pattern in the female cohort and the male cohort (Fig. 1a–d).

### FRAX assessment in LSS patients

The FRAX questionnaire revealed that 28 of the 65 LSS patients had a moderate to high risk (> 10%) of having an osteoporotic fracture within 10 years, and 37 of the 65 were assessed as having a low fracture risk (≤ 10%). Adding the measurement of FN BMD to FRAX, which has been suggested to give a more accurate prediction [21, 25], 25 of the 65 LSS patients were assessed to be moderate- to high-risk patients.

In the LSS group, 46 of the 65 patients had at least one vertebra (lateral spine measurement) with a *T*-score of ≤ − 2.5. Considering the FRAX questionnaire results for these 46 patients, only 50% were assessed to run a moderate to high risk of fracture within 10 years. With a combination of FRAX and FN BMD, 24 of the 46 were assessed as being moderate- to high-risk patients (Table 6).

**Table 6:** FRAX assessment in LSS patients

*T*-score lateral	FRAX		FRAX + FN BMD		FN *T*-score	
	Moderate/high risk	Low risk	Moderate/high risk	Low risk	≤ − 2.5	> − 2.5
≤ − 2.5	23	23	24	22	10	36
> − 2.5	5	14	1	18	–	19

Evaluation of the risk of having an osteoporotic fracture within 10 years, using FRAX alone or FRAX with FN BMD and subdivided according to FN *T*-score. The patients (*n* = 65) were divided into two different groups: *T*-score in the lateral projection ≤ − 2.5 and *T*-score in the lateral projection > − 2.5, measured in at least one vertebra. Moderate to high risk (> 10% risk) and low risk (≤ 10% risk) of having an osteoporotic fracture within 10 years

In the 19 patients without any vertebrae with a *T*-score of ≤ − 2.5, 14 patients were classified as having a low fracture risk by FRAX alone. When FRAX was used in combination with FN BMD, 18 of the 19 were classified as being low-risk patients.

This means that when using lateral spine *T*-score measurement as the “truth” for diagnosing the poor bone quality of the spine, FRAX alone had a specificity of 74% and a sensitivity of 50%, while FRAX combined with BMD FN had a specificity of 95% and a sensitivity of 52%.

Instead of using mid-lateral spine *T*-score measurement as the “truth,” FRAX alone had a specificity of 70% and a sensitivity of 60%, while FRAX combined with BMD FN had a specificity of 60% and a sensitivity of 49%.

## Discussion

In the present study, the occurrence of a *T*-score ≤ − 2.5 in LSS patients varied between a few percents up to 50%, depending on the projection and location of the DXA measurement. No major differences in the spine or hip DXA measurements were observed in the LSS and HOA patients. Furthermore, by using the FRAX questionnaire in combination with an available DXA of the FN, only 52% of the patients with a *T*-score ≤ − 2.5 of the spine (measured by lateral spine DXA) could be identified.

The proportion of patients with osteoporosis/osteopenia in this study who required spinal stenosis surgery was in accordance with previous reports. Andersen et al. found 9% osteoporosis and 30% osteopenia using the lumbar AP view in LSS patients (of both genders) [26]. Lee et al. found 22.6% osteoporosis and 56.6% osteopenia in LSS patients over 60 years (in the AP view), which was considerably higher than in our study. They investigated LSS patients who did not require surgery [27], which suggests that their patients may have differed from ours in several ways, e.g. higher age and co-morbidities.

Chin et al. examined patients requiring all types of spinal surgery. They found bone density prevalence that was in line with our findings in LSS patients using the lateral lumbar view measurements; 41% of the women had osteoporosis and 46% had osteopenia, [9] as compared to 49% and 38% in our study.

It is well known that the DXA method gives different results for BMD depending on location and projection. As a consequence, the number of patients who are classified as being osteoporotic can vary widely depending on the type of measurement used. Lumbar AP and FN are the most commonly used projections for measurement of BMD [17]. In the present study, FN measurements led to the classification of rather few LSS patients as being osteoporotic. We also found that the proportion of patients (both men and women) with LSS who had a *T*-score ≤ − 2.5 ranged as much as between 4% for the AP projection (classified as being osteoporotic) and 49% for the lat-mid measurement. One of the reasons for using lateral spine DXA measurements is that osteophytes are very common in the lumbar spine. This may result in “falsely” high BMD values when the AP spine projection is used.

A lower BMD, and therefore a higher incidence of osteoporosis, was seen in the LSS group for the upper/mid lumbar vertebrae than for the lower lumbar vertebrae, which may be explained by more degenerative changes and higher sclerosis in the lower lumbar segments, where spinal stenosis problems are most frequent. This distribution was also seen in the HOA group, indicating that this is not specific to LSS patients but rather reflects the BMD distribution in this age group. Degenerative lumbar spine changes are also frequent in individuals without LSS symptoms. However, for the lowest vertebrae (L4) the mean AP BMD was higher in LSS patients than in HOA patients and the opposite was true for the mean FN BMD, which was slightly higher in the HOA group. This probably reflects the osteophytes and sclerosis in the area of symptoms for the two patient groups and is supported by the fact that L4–L5 is the most common level for spinal stenosis.

To identify the presence of poor bone quality in patients who require spine surgery is important, especially if instrumentation is to be performed and the knowledge of a poor BMD may influence what surgical method that should be chosen. DXA measurement is time-consuming and uses up resources. There are alternative measurement methods to DXA that can be used for BMD measurements such as quantitative computed tomography and DensiProbe Spine [28]. These methods are less validated and used compared to DXA; however, these may be more sensitive methods [22]. However, also, these require equipment and personnel resources.

In the present study, we investigated whether the FRAX tool, which is a quick and easy tool used in the assessment of fracture risk and not requiring any specific equipment, would be of any benefit in identifying LSS patients with lumbar osteoporosis. Quite often, these patients already have been screened for osteoporosis with FN DXA, which then could be added to the FRAX questionnaire. By using FRAX in combination with an FN DXA, we were able to identify 52% of the LSS patients with a *T*-score ≤ − 2.5 of the spine, as evaluated by a lateral spine DXA measurement. Using FRAX alone, we could identify 50%.

The use of FRAX alone or in combination with FN DXA to identify patients with poor lumbar bone quality in the work-up procedure for surgery can therefore be considered to be of doubtful value. However, FRAX is still of value for a general assessment of future fracture risk [29].

The main limitations of the present study were the limited number of LSS patients included, despite the inclusion of patients over several years. This was due to the fact that many of the patients at our university clinic presented with high comorbidity and/or previous surgery and could therefore not be included. However, few previous studies have compared the different projections of hip and lumbar DXA measurements in LSS patients.

## Conclusion

In conclusion, the study highlights the difficulties in assessing and defining a poor bone quality and osteoporosis. Most previously, healthy patients requiring surgery for spinal stenosis had *T*-score values ≤ − 2.5 in the lateral view of the lumbar spine. However, large variations in BMD between different projections and locations were apparent. The FRAX instrument showed low sensitivity and specificity in identifying reduced bone quality in the lumbar spine. It may therefore be important to preoperatively evaluate the lateral lumbar BMD specifically in LSS patients in order to optimize treatment strategies.

## Abbreviations

AP: Anterior-posterior
BMD: Bone mineral density
DXA: Dual-energy X-ray absorptiometry
FN: Femoral neck
FRAX: Fracture risk assessment tool
HOA: Hip osteoarthritis
LSS: Lumbar spinal stenosis
QCT: Quantitative computed tomography
WHO: World Health Organization
